# Microglial repopulation alleviates age-related decline of stable wakefulness in mice

**DOI:** 10.3389/fnagi.2022.988166

**Published:** 2022-10-03

**Authors:** Hanxiao Liu, Mohamed Badawy, Shaoqin Sun, George Cruz, Shaoyu Ge, Qiaojie Xiong

**Affiliations:** Department of Neurobiology and Behavior, Stony Brook University, Stony Brook, NY, United States

**Keywords:** microglia, microglia repopulation, aging, stable wakefulness, sleep

## Abstract

Changes in wake/sleep architecture have been observed in both aged human and animal models, presumably due to various functional decay throughout the aging body particularly in the brain. Microglia have emerged as a modulator for wake/sleep architecture in the adult brain, and displayed distinct morphology and activity in the aging brain. However, the link between microglia and age-related wake/sleep changes remains elusive. In this study, we systematically examined the brain vigilance and microglia morphology in aging mice (3, 6, 12, and 18 months old), and determined how microglia affect the aging-related wake/sleep alterations in mice. We found that from young adult to aged mice there was a clear decline in stable wakefulness at nighttime, and a decrease of microglial processes length in various brain regions involved in wake/sleep regulation. The decreased stable wakefulness can be restored following the time course of microglia depletion and repopulation in the adult brain. Microglia repopulation in the aging brain restored age-related decline in stable wakefulness. Taken together, our findings suggest a link between aged microglia and deteriorated stable wakefulness in aged brains.

## Introduction

Sleep and wake are two basic brain states necessary for the health of all living organisms, while the stability of sleep architecture is vulnerable to the aging process. Age-related changes in sleep/wake behavior are mainly manifested by sleep fragmentation during the rest phase and frequent drowsiness during the active phase in humans and rodents (Mander et al., 2017; McKillop and Vyazovskiy, 2020). Changes in sleep architecture in turn indicate accelerated brain aging or pathological conditions, such as dementia (Carvalho et al., 2017; Sabia et al., 2021; Gao et al., 2022). Sleep studies in the mouse model reported that aged mice (>1 year old) showed a dramatic change in sleep architecture (Hasan et al., 2012; Wimmer et al., 2013; McKillop et al., 2018; Li et al., 2022), while the details about progressive changes at different ages are less addressed.

Altered microglia functions were observed under both healthy aging and neurodegenerative conditions, such as Alzheimer’s disease (Grabert et al., 2016; Niraula et al., 2017; Candlish and Hefendehl, 2021). Microglia are the resident immune cells within the brain. In addition to its crucial role in the inflammation process, microglia actively survey the brain parenchyma and control multiple physiological functions (Hanisch and Kettenmann, 2007; Prinz et al., 2019), such as synapse formation and pruning (Kettenmann et al., 2013), neuronal excitability and blood flow (Badimon et al., 2020; Cserép et al., 2020; Bisht et al., 2021). There is a growing body of evidence suggesting that transcriptional, morphological, and functional changes of microglia with aging (Grabert et al., 2016; Niraula et al., 2017; Olah et al., 2018; Hammond et al., 2019). For instance, inflammatory and interferon-responsive microglia emerge in aged mouse brain (Hammond et al., 2019). Alterations of microglial properties, with increasing age, provide the basis for the onset of age-related decay of many central nervous system-related physiological and cognitive functions (Giorgetti et al., 2019). However, it is unknown whether changes in microglia serve as a potential mechanism for alteration of sleep architecture along aging.

Sleep/wake behavior is tightly correlated with microglia activity. Change in brain states (wake/anesthesia) induces an alteration of microglia morphology (Liu et al., 2019; Stowell et al., 2019), and sleep deprivation elevates the activity of microglia by reducing microglial process complexity and elevating pro-inflammatory cytokines production (Hsu et al., 2003; Wadhwa et al., 2017; Tuan and Lee, 2019). A recent study further suggests that microglia actively control sleep architecture by modulating the stability of wakefulness (Liu et al., 2021). Although microglia are a type of long-lived brain cell, they maintain a high renewal capability at any examined age (Elmore et al., 2014, 2015; Bruttger et al., 2015; Fuger et al., 2017; O’Neil et al., 2018). Previous studies suggest renewing of microglia population *via* microglia depletion not only rescues neurogenesis and neuronal morphologies in the aged mouse brain, but also alleviates cognitive deficits following brain injury (Elmore et al., 2018; Willis et al., 2020). Whether global renewal of microglial population in the aging brain is beneficial to the decay of sleep functions is still unclear.

By systematically examining sleep architecture and microglia morphology across multiple sleep/wake controlling brain regions in mice at different ages, we found that aging progressively changed the stability of wakefulness and microglial morphology which is prominent as early as 6 months in mice. We further revealed that early experience of microglia repopulation during adulthood alleviates decay of stable wakefulness in older age. All together, our results indicate that the stability of wakefulness is vulnerable to aging, and can be rescued by microglial repopulation during adulthood.

## Results

### Aging mice display declined stable wakefulness at nighttime

**FIGURE 1:**
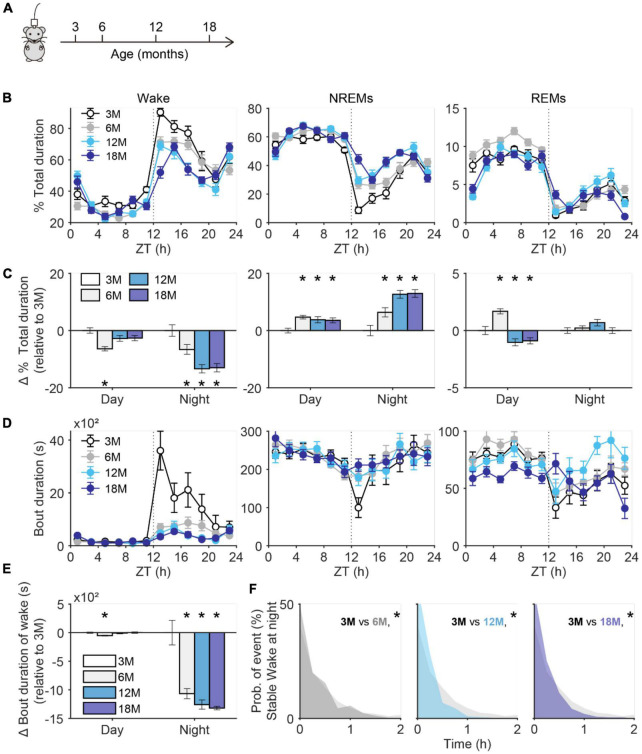
Decay of stable wakefulness in aging mice. **(A)** The schematic figure shows electroencephalogram/electromyography (EEG/EMG) recordings were performed in mice at 3, 6, 12, and 18 months old. **(B)** Percent of total duration spend on wake (left), NREMs (non-rapid eye movement sleep, middle), and REMs (rapid eye movement sleep, right) for 24 h recording. Data were analyzed every 2 h. 3M, *N* = 16 recordings from 4 mice; 6M, *N* = 20 recordings from 4 mice; 12M, *N* = 16 recordings from 4 mice; 18M, *N* = 20 recordings from 4 mice. **(C)** Change in total duration spend on wake (left), NREM (middle), and REM (right) sleep during daytime and nighttime. All values were normalized to data collected from 3 months old mice. **(D)** Bout duration of wake (left), NREM (middle), and REM (right) sleep for 24 h recording. Data were analyzed every 2 h. **(E)** Change in bout duration of wake at day and night. All values were normalized to data collected from 3 months old mice. **(F)** Comparison of bout duration for stable wakefulness at night between mice at age of 3 months and older age. *p < 0.05; two-sided unpaired *t*-test for panels **(C,E)**; two-sided Kolmogorov–Smirnov test for panel **(F)**.

To investigate the potential link between microglia and wake/sleep changes in aging, we first systematically determined the changes in wake/sleep architecture in aging mice. Using continuous electroencephalogram (EEG) and electromyography (EMG) recordings (24 h per session) in mice, we compared their wake and sleep behaviors among four different ages: 3 months (3M), 6 months (6M), 12 months (12M), and 18 months (18M) (Figure 1A). We found that older mice spent less time awake when compared to 3-month-old ones, particularly during nighttime (12–24 Zeitgeber Time, their active time as nocturnal animals) (Figures 1B,C). Starting from 6-months-old, mice had gradually increased non-rapid eye movement sleep (NREMs) with the cost of decreased wake time, but the change in rapid eye movement sleep (REMs) was less consistent between day and night (Figure 1C). REMs was decreased during the day and increased during the night in aged mice (Figure 1C), which is consistent with previous studies (Wimmer et al., 2013; McKillop et al., 2018). We further found that nighttime bout numbers of wake and NREMs increased in aging mice, with less consistent changes in REMs (Supplementary Figures 1A,B). Interestingly, the bout durations of wake state dramatically decreased specifically in the long-duration range (>1 h) (Figures 1D–F), whereas there was no consistent alteration in bout durations of NREMs and REMs (Figure 1D; Supplementary Figure 1C). These findings suggest that stable wakefulness gradually declined in aging mice.

### Aging mice have more microglia with decreased length of processes

**FIGURE 2:**
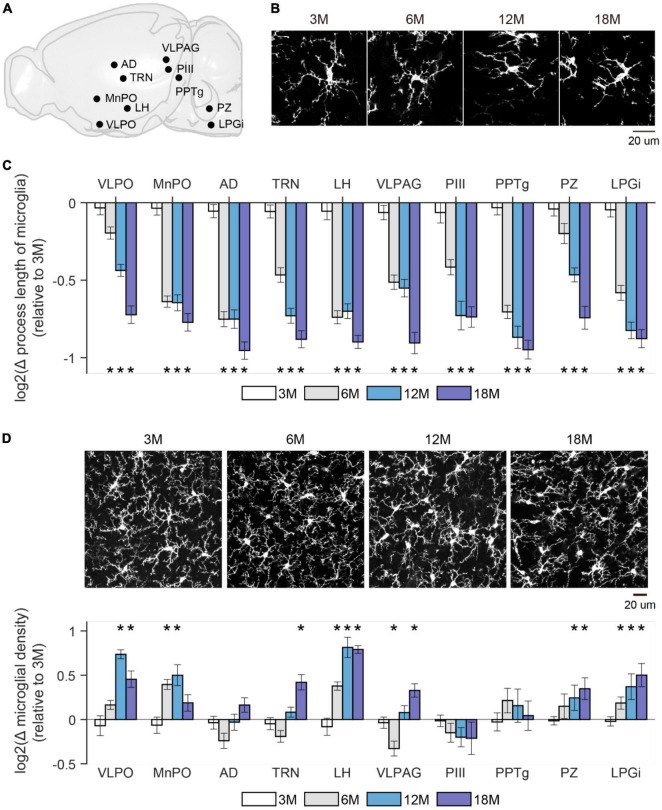
Microglial morphology changed in aging mice. **(A)** A sagittal brain image showed sleep/wakefulness-related brain regions being examined. VLPO, Ventrolateral preoptic area; MnPO, median preoptic area; AD, anterodorsal thalamus; TRN, thalamic reticular nucleus; LH, lateral hypothalamus; VLPAG, ventrolateral periaqueductal gray; PIII, perioculomotor region of midbrain; PPTg, pedunculopontine tegmental nucleus; PZ, parafacial zone; LPGi, lateral paragigantocellular nucleus. **(B)** Representative images of microglia were collected from the TRN region in mice at indicated ages. **(C)** Change in total process length of microglia in above-mentioned brain regions from mice at the indicated age. The total process length of microglia showed a progressive decrease in all examined brain regions with increasing age. All values were presented by log2 of ratio relative to data collected from 3 months old mice. *p*-value was obtained by comparing the value between 3 months and older ages. 3M, 6 mice; 6M, 6 mice; 12M, 6 mice; 18M, 2 mice. **(D)** Change in density of microglia in above-mentioned brain regions from mice at the indicated age. Microglia density showed a trend of increase across the whole brain. Representative images of microglia were collected from the LH in mice at indicated ages. All value was presented by log2 of ratio relative to data collected from 3 months old mice. *p*-value was obtained by comparing the value between 3 months and older ages. 3M, 6 mice; 6M, 7 mice; 12M, 6 mice; 18M, 4 mice. *p < 0.05; two-sided unpaired *t*-test for panels **(C,D)**.

It’s widely recognized that the morphological change of microglia faithfully reflects their altered physiological functions, such as increased immune response of de-ramified microglia (Nimmerjahn et al., 2005; Prinz et al., 2019). To determine microglial differences in aging mice, we prepared brain slices from four age groups (3M, 6M, 12M, and 18M) of CX3CR1^*Cre*–*ERT*2–*EYFP*/+^ mice in which microglia are labeled with enhanced yellow fluorescence protein (EYFP) (Parkhurst et al., 2013; Liu et al., 2021). To confirm the cell identity, we also performed immunostaining with a microglia marker, ionized calcium binding adaptor molecule 1 (Iba1) (Ito et al., 1998). Cells co-labeled with Iba1 and EYFP were considered microglia. We quantified the densities and total process length of microglia in various brain regions that have been linked to wake/sleep regulations (Scammell et al., 2017; Liu and Dan, 2019), including ventrolateral preoptic area (VLPO), median preoptic area (MnPO), anterodorsal thalamus (AD), thalamic reticular nucleus (TRN), lateral hypothalamus (LH), ventrolateral periaqueductal gray (VLPAG), perioculomotor region of the midbrain (PIII), pedunculopontine tegmental nucleus (PPTg), parafacial zone (PZ), and lateral paragigantocellular nucleus (LPGi) (Figures 2A,B). We found that compared to brains from 3 months old mice, microglia in aging brains had dramatically decreased total process lengths across all the examined brain regions (Figure 2C), and most of the examined brain regions showed a gradual increase of microglial density with increasing age, such as VLPO, MnPO, LH, PZ, and LPGi (Figure 2D). These age-related differences in microglia have a similar timeline as that of stable wakefulness (Figure 1), suggesting a potential link between them.

### Microglia depletion and repopulation changed stable wakefulness in adult mice

**FIGURE 3:**
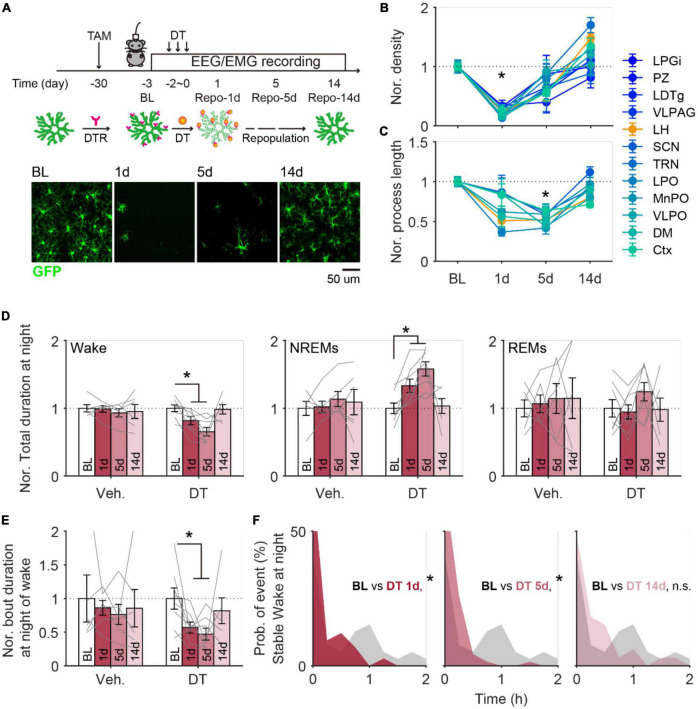
Microglia depletion induced changes in sleep architecture recovered along with microglial repopulation in adult mice. **(A)** Schematic image showing the timeline for microglia depletion and subsequent repopulation. EEG/EMG signals were continuously recorded in mice at 1 day before depletion onset (baseline, BL) and 1 (1d), 5 (5d), and 14 days (14d) after depletion. Representative images were collected from cortex. DT, diphtheria toxin; DTR, diphtheria toxin receptor. Scale bar, 20 μm. **(B)** Microglia density decreased to the lowest level at 1 day after depletion and globally recovered back at 14 days later. Ctx, cortex; DM, dorsomedial hypothalamic nucleus; VLPO, ventrolateral preoptic area; MnPO, median preoptic area; LPO, lateral preoptic area; TRN, thalamic reticular nucleus; SCN, suprachiasmatic nucleus; LH, lateral hypothalamus; VLPAG, ventrolateral periaqueductal gray; LDTg, laterodorsal tegmental nucleus; PZ, parafical zone; LPGi, lateral paragigantocellular nucleus. BL, 6 mice; Repo-1d, 6 mice; Repo-5d, 5 mice; Repo-14d, 4 mice. **(C)** The total process length of microglia decreased to the lowest level at day 5 and globally recovered back at day 14. **(D)** The normalized total duration of wake (left), NREMs (middle), and REMs (right) at nighttime in mice with vehicle (Veh.) or DT injections. All data was normalized to data from BL. Sleep architecture showed a dramatic change at the time with 1–5 days of repopulation and recovered back at repopulation for 14 days. Veh., 5 mice; DT, 5 mice. **(E)** The normalized bout duration of wake (left), NREMs (middle), and REMs (right) at nighttime in mice with vehicle (Veh.) or DT injections. All data was normalized to data from BL. **(F)** Distribution of duration for stable wakefulness at night between BL and day 1 (left), day 5 (middle), and day 14 (right) in mice with microglia depletion. *p < 0.05, two-sided unpaired *t*-test for panels **(B,C)**; two-sided paired *t*-test for panels **(D,E)**; two-sided Kolmogorov–Smirnov test for panel **(F)**.

To determine whether the age-related changes of microglia play any role in wake/sleep behaviors in aging mice, we next manipulated microglia and assessed the impacts on wake/sleep behaviors. Using the transgenic mice (CX3CR1^*Cre*–*ERT*2–*EYFP*/+^:R26*^iDTR/+^*) in which expression of diphtheria toxin (DT) receptor is controlled by CX3CR1 promoter in a Cre-dependent manner (Parkhurst et al., 2013; Liu et al., 2021), we first induced microglia depletion in young adult mice (3 months old) and track their wake/sleep changes along with the depletion and repopulation of microglia. We delivered tamoxifen (gavage) to induce DT receptor expression in microglia, 4 weeks later we systematically injected DT for 3 consecutive days to specifically deplete microglia through the DT receptors (Parkhurst et al., 2013; Bruttger et al., 2015; Liu et al., 2021). We found ∼70% reduction of microglia from the whole brain (Figures 3A,B), while other CX3CR1-expressing immune cells in peripheral systems were less affected according to our previous finding (Liu et al., 2021). All examined brain regions showed the lowest microglial density (*p* < 0.05) on day 1 and reached the lowest total process length (*p* < 0.05) on day 5 (Figures 3B,C). On day 14, both density and morphology of microglia showed a full recovery except over-repopulated microglia density observed in TRN (170 ± 12.6%, *p* = 0.005) and LH (148 ± 7.7%, *p* = 0.0028), and shorter total process length observed in cortex (71 ± 3.6%, *p* < 0.001), VLPO (80 ± 5.2%, *p* = 0.017), and LH (80 ± 4.9%, *p* = 0.004) (Figures 3B,C).

From EEG/EMG analyses, we observed significantly decreased wake durations and increased NREMs durations on day 1 and day 5 during nighttime (Figure 3D), and no clear difference during daytime (Supplementary Figure 2A). At night, both bout duration of wake and stable wakefulness showed a dramatic decline on day 1 and day 5 (Figures 3E,F). These wake/sleep changes also recovered by day 14, consistent with the timeline found in microglia following the DT injection (Figures 3D–F). Control mice that received tamoxifen treatment and vehicle injection (instead of DT) showed relatively stable sleep/wake behaviors throughout the same recording period (Figures 3D,E; Supplementary Figure 2B).

Our previous study revealed that microglia modulated stable wakefulness in adult mice (Liu et al., 2021). The current findings of decreased microglia process length and decreased stable wakefulness from both aging brains and microglia-depleted brains, imply that the alterations of microglia in the aging brain may be one of the causes of altered wake stability.

### Microglia repopulation partially rescued age-related decline of stable wakefulness

**FIGURE 4:**
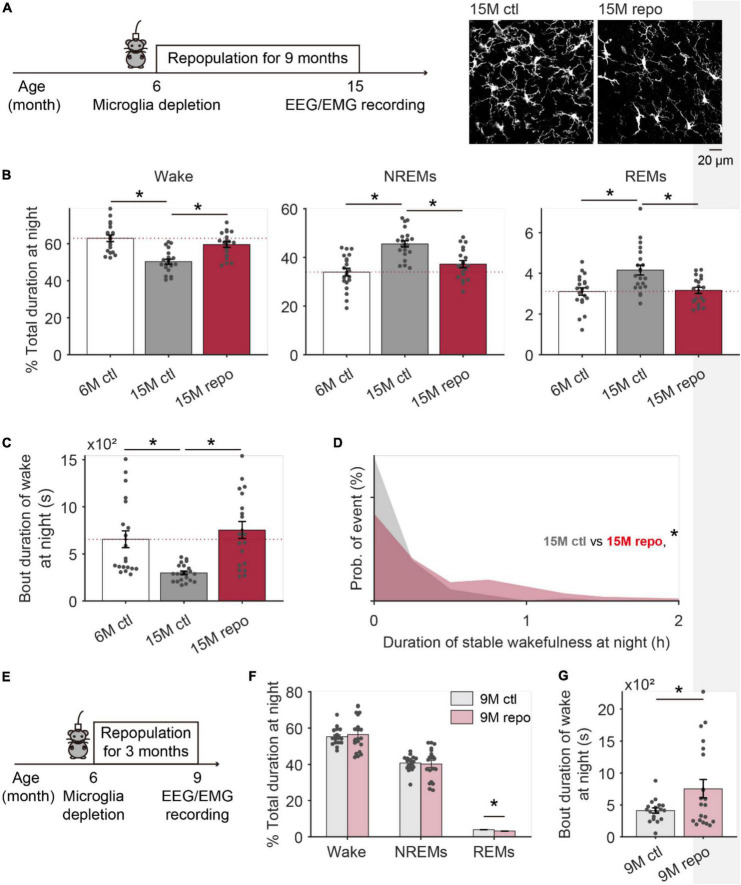
Aged mice with repopulated microglia during adulthood displayed improved wake stability at night. **(A)** The schematic image showing EEG/EMG recording was performed in 15 months old mice with 9 months of microglia repopulation. Representative images are microglia collected from the TRN of mice with (15M repo) or without early repopulation (15M ctl). **(B)** The total duration of wake (left), NREMs (middle), and REMs (right) at nighttime in mice with microglia repopulation (15M repo, N = 20 recording from 5 mice), age-matched control mice (15M ctl, *N* = 21 recording from 5 mice) and 6 months old control mice (6M ctl, *N* = 20 recordings from 4 mice). **(C)** Bout duration of wakefulness at night for each group. **(D)** Compared to age matched controls, stable wakefulness at night has been significantly improved in mice with early experience of microglial repopulation. **(E)** The schematic image showing EEG/EMG recording was performed in 9 months old mice with 3 months of microglia repopulation. **(F)** The total duration of wake NREM, and REM sleep at nighttime in mice with microglia repopulation (9M repo, *N* = 20 recording from 4 mice), age-matched control mice (9M ctl, *N* = 18 recording from 4 mice). **(G)** Bout duration of wakefulness at night for 9-month-old mice with/without repopulation. *p < 0.05; one-way ANOVA with Fisher’s *post hoc* test for panels **(B,C)**; two-sided Kolmogorov–Smirnov test for panel **(D)**; two-sided unpaired *t*-test for panels **(F,G)**.

After depletion, the repopulated microglia demonstrate similar morphology and functions as the original resident microglia (Bruttger et al., 2015; Elmore et al., 2015, 2018), and promote brain repair in pathological conditions (Rice et al., 2017; Henry et al., 2020; Willis et al., 2020). If the decline of stable wakefulness in the aged brain is due to altered microglia activity during aging, depleting microglia and allowing them to repopulate might improve the deteriorated stable wakefulness. To test this hypothesis, we used the same strategy described in Figure 3A to deplete microglia in 6-month old CX3CR1^*Cre*–*ERT*2–*EYFP*/+^:R26*^iDTR/+^* mice, and examined the wake/sleep behaviors 9 months later at 15-month old (15M repo) (Figure 4A). Compared to the 6 months old control mice (6M ctl), 15M control mice had decreased wake and increased NREMs durations at nighttime (Figure 4B), consistent with our findings in Figure 1. Notably, 15M mice with early experience of microglia depletion and repopulation (15M repo) displayed higher wake total durations and bout durations. In addition, there was lower NREMs total durations in the 15M repo than the 15M control mice, which is closer to the conditions in 6M control mice (Figures 4B,C). Moreover, the 15M repo mice had recovered stable wakefulness when compared to the 6M control and 15M control mice (Figure 4D). With further morphological analysis, we found that microglia in 15M repo mice showed decreased density, and comparable total process length across all examined brain regions (Supplementary Figure 3). The decreased microglial density in aged mice with repopulation may indicate a rejuvenated micro-environment within the brain.

To further test whether 9 months of repopulation is necessary to observe the recovery of age-related decay on stable wakefulness, we depleted microglia from a new cohort of 6-month-old mice, and sleep architecture was recorded 3 months later (9M repo, Figure 4E). Compared to age-matched control mice in 9-month-old (9M ctl), total duration for wake and NREMs in 9M repo mice was comparable (Figure 4F), but the bout duration of wake in 9M repo mice was dramatically extended (Figure 4G). It indicates a gradual prominent effect of early microglia repopulation on decline of stable wakefulness with increasing age.

Altogether, our findings indicated that microglia repopulation in adulthood is beneficial to slow down the decay of stable wakefulness in aged mice.

## Discussion

In this study, we found a progressive decline of stable wakefulness, and changes in microglial morphology in aging mice can be as early as from 6-month-old. Acute microglia depletion in young adult mice strongly impaired the stable wakefulness which was restored along with the repopulation of microglia. Aged mice with microglia depleted and repopulated early in adulthood had less decline of stable wakefulness. Taken together, our results indicate that microglia play a role in regulating wake/sleep behavior and repopulation may alleviate the decay of stable wakefulness in aged mice.

The decay of multiple physiological systems manifests functional aging at different rates with increasing age. In the rodent model of aging, the decline in physical activities and deficits in visual/auditory systems start from 6 months after birth, while cognitions decline beginning at age of 12 months (Radulescu et al., 2021; Yanai and Endo, 2021). Aging dramatically altered sleep/wake behavior characterized by excessive sleep at the active phase and sleep fragmentation at the rest phase in both rodents and humans (Mander et al., 2017). Altered microglia function has been correlated with the decay of many central nervous system-related physiological functions along with aging (Niraula et al., 2017; Candlish and Hefendehl, 2021). In this study, we found that microglia in aged mice contribute to the decline of stable wakefulness (excessive sleep) during the active phase. In young adult mice, our previous study showed that microglia modulate stable wakefulness through TRN neurons (Liu et al., 2021). Additional work is required to assess how aging affects TRN neuronal activity and whether the age-related decline of stable wakefulness is also due to the vicissitude in TRN.

Microglial properties display a regional specificity in terms of morphology, density, and transcriptional profile (Lawson et al., 1990; Grabert et al., 2016; Ayata et al., 2018). Therefore, it’s reasonable to observe the different regional responses of microglia under certain circumstances (i.e., aging). Although aging induced a comparable decrease in process length of microglia across the whole brain, the cell density of microglia showed a regional difference with the aging process. Relative to microglia in the thalamus (AD, TRN) and midbrain (PIII, VLPAG), microglia located in the brain stem (PZ, LPGi) and hypothalamus (VLPO, MnPO, LH) showed more dramatic proliferation with increasing age. Although the whole brain microglia complied with a comparable timeline for repopulation following global depletion, lateral hypothalamus microglia showed an over-repopulation and dramatic morphological difference from original microglia following 14 days of repopulation (Figures 3A–C). Mounting evidence suggests that lateral hypothalamus-related neural circuits play an essential role in wake and sleep regulation, even in aged mice (Hassani et al., 2009; Jego et al., 2013; Herrera et al., 2016; Li et al., 2022). Repopulated microglia in lateral hypothalamus may potentially contribute to the recovery of declined stable wakefulness in aging mice. Moreover, sexual dimorphism of microglia also exhibits a region-specific manner (Han et al., 2021). Further work is required to assess the potential effects of microglial sexual dimorphism on the aging-related microglial and brain vigilance alterations observed in this study.

Microglia repopulation is a progressive process, with the cell density recovered faster than morphology according to our observation (Figures 3A–C) and previous studies (Elmore Monica et al., 2014; Salter Michael and Beggs, 2014; Bruttger et al., 2015; Elmore et al., 2015). Five days after the depletion, most brain regions showed a dramatic recovery in microglia density, whereas the total process length of microglia decreased to the lowest level (Figure 3C). Microglia morphology is tightly correlated with its activity level. The dramatic difference in morphology of newly repopulated microglia may reflect changed inflammatory levels within the brain, such as interleukin 1 (Bruttger et al., 2015), which represents one important factor in modulating sleep (Opp and Krueger, 1991; Takahashi et al., 1997; Imeri and Opp, 2009). Astrocytes also play an important role in sleep regulation (Bojarskaite et al., 2020; Vaidyanathan et al., 2021). According to earlier studies, there is astrogliosis during the repopulation process (Bruttger et al., 2015), which may contribute to increased NREMs during the repopulation process. On day 14, both morphology and density of microglia recover back to baseline level, and we observed the concurrent recovery of sleep architecture in those mice with completed microglia repopulation. Whether astrocyte density and activity back to normal range needs to be addressed in the future.

Aging/neurodegeneration-related functional decay of microglia primarily plays a neurotoxic role to the brain functions due to augmented inflammatory response and impaired housekeeping functions (Niraula et al., 2017). Selective accumulation of alpha-synuclein in microglia leads to progressive degeneration of dopaminergic neurons (Bido et al., 2021), and microglia-mediated plaque-driven loss of perineuronal nets and subsequent reduction of parvalbumin-positive interneurons in Alzheimer’s disease mouse model (Crapser et al., 2020). A growing body of evidence suggest that manipulation of microglia would benefit age-related physiological conditions. For example, microglia depletion decreased age-related loss of motor units (Giorgetti et al., 2019), and sustained microglia depletion blocks plaque formation in 5xFAD mouse model of Alzheimer’s disease (Sosna et al., 2018; Spangenberg et al., 2019). Repopulating microglia in old age attenuated cognitive deficits in aged mice and mice with traumatic brain (Elmore et al., 2018; Willis et al., 2020), which may be mediated by partially reversing age-related alteration in the transcriptional profile of microglia and neuronal functions (Elmore et al., 2018; O’Neil et al., 2018). Our study further suggests that early experience of microglial repopulation at the beginning of alterations in stable wakefulness ameliorates decay of stable wakefulness at old ages. As such, renewing of microglia niche in the aged brain with repopulation serve as a potential possibility for clinical treatment of age-related decay of physiological and cognitive functions. Whether there is any difference in the beneficial effect of microglia repopulation onset at middle age or older age needs further exploration.

Microglia in healthy aging processes have been described as dystrophic and senescent (Streit et al., 2004; Angelova and Brown, 2019), with altered transcriptional and secretory profiles (Sierra et al., 2007; Hammond et al., 2019; Sala Frigerio et al., 2019). Our current study suggests that aged microglia may contribute to the declined stable wakefulness in aged mice, but the molecular underpinnings remain unknown. Our previous study revealed ceramide, a bioactive metabolite, mediates microglia modulation on stable wakefulness (Liu et al., 2021). Earlier studies indicate that ceramide progressively accumulates in the brain with increasing age (Cutler et al., 2004; Sacket et al., 2009), and acutely increased brain ceramide level impairs stable wakefulness (Liu et al., 2021). Therefore, ceramide might be a candidate for mediating the altered stable wakefulness in aging. How microglia regulates ceramide concentration in young and aging brains, and whether the early experience of microglia repopulation has effects on age-related accumulation of ceramide needs further exploration.

## Materials and methods

### Animal

Wild-type C57BL/6J (11 males 9 females), CX3CR1^CreERT2–EYFP/+^ mice (12 males 10 females) with comparable number of both sex at age of 3, 6, 12, and 18 months old, and CX3CR1^CreERT2–EYFP/+^:R26^iDTR/+^ mice with comparable number of both sex at age of 3 (13 males 12 females), 6 months old (12 females 6 males) were used for the experiments. Due to limitations in collecting aged mice, only 2 mice at age of 18 months old were used for studying age-related changes in microglia morphology. We validated that the trend for age-related change for both mice is comparable. Mice were group-housed with 2–4 animal per cage and entrained to a 12-h/12-h light/dark cycle with 17–26°C and 30–70% humidity, food and water available *ad libitum*. All procedures were approved by the Stony Brook Animal Care and Use Committee and carried out in accordance with the National Institutes of Health standards.

### Surgery

Adult mice were anesthetized with isoflurane (3% for induction followed by 1% for maintenance) and placed in a stereotaxic apparatus. Both eyes were covered with a layer of ointment during surgery. Implantation of EEG/EMG channel was consistent with our previous work (Liu et al., 2021). After shaving and sterilizing the skin, the scalp was incised to expose the skull. Connective tissue was gently removed with a scalpel, and a thin layer of iBond Self Etch was applied. Four holes were drilled into the skull at the following coordinates: 1st EEG channel: AP: +2 mm, ML: +1.5 mm; 2nd EEG channel: AP: –4 mm, ML: +1.5 mm, reference channel (AP: +2 mm, ML: –1.5 mm), and ground channel site (AP: –4 mm, ML: –1.5 mm). A 2-EEG/1-EMG headmount (8201, Pinnacle Technology Inc.) was aligned to the craniotomy sites and pre-fixed to the skull with a small drop of dental cement. Four EEG screws (0.10-in EEG screws for the anterior craniotomy sites and 0.12-in EEG screws for the posterior craniotomy sites) were inserted into the corresponding channels of the headmount and screwed to the skull. 2 EMG leads were inserted on each side of the neck muscles to record postural tone. All implants were secured to the skull with dental cement. Following surgery, mice were kept on a heating pad and closely monitored until they were fully awake, and then housed individually post-surgery. Mice were allowed to recover for at least 2 weeks before the next step.

### EEG/EMG recordings and data analysis

Implanted mice were habituated to the tethered recording system in their home cage for at least 3 days. The recording was performed for 24 h in the animal’s home cage with food and water available *ad libitum*. All cage maintenance work was performed after each daily recording session was completed.

Implanted mice were tethered to a preamplifier (8202-SL, Pinnacle Technology, Inc.) and a commutator (8204, Pinnacle Technology, Inc.) for free-moving recordings. Signals were collected with a 100 × gain, 0.5-Hz high-pass filter and 400 Hz sampling rate using Sirenia Acquisition software (Pinnacle Technology, Inc.) and analyzed with Sirena sleep software (Pinnacle Technology, Inc.). Simultaneous video recording was performed to monitor animal behavior. The recording was performed for 24 h and repeated 5 times every other day for each animal except mice under the process of microglia depletion and repopulation. Brain state scoring was first processed using the cluster scoring feature of Sirenia Sleep, and then manually re-inspected epoch-by-epoch based on the frequency and amplitude signatures of the signals, and corresponding behaviors on video. States were assigned based on consecutive, non-overlapping, 4-s windows as either wakeful, NREM sleep, or REM sleep using scoring criteria described previously (Liu et al., 2021). Because certain behavior may introduce a distinct power spectrum during the wake state, such as theta oscillation during running (Kropff et al., 2021), the power spectrum of wake state in behaving mice showed a less clear dominant frequency band as NREMs (0.5–4 HZ) or REMs (6–9 HZ). Therefore, following the criteria used in reported studies, we defined wake state in mice by low-amplitude desynchronized EEG signal with high EMG activity. Synchronized EEG with high-amplitude low-frequency (0.5–4 Hz) EEG activity and low EMG activity were defined as NREM sleep; and prominent theta frequency (6–9 Hz) EEG and low EMG activity were defined as REM sleep. Proportions (%) for total duration, bout duration, and bout number/h for each brain state were calculated. Wakeful bouts longer than 2 min were defined as stable wakefulness.

### Drug applications

We used a CX3CR1^CreERT2/+^:R26^iDTR/+^ transgenic mouse line for global microglial depletion. Mice were administered tamoxifen (T5648, Sigma) to initiate DT receptor expression (100 mg/kg, gavage twice, with 2-day intervals) at 4 weeks before depletion onset. Microglia labeled with DT receptor were depleted following i.p. injections of DT (D0564, Sigma) for 3 consecutive days (25 μg/kg).

### Histology

Mice were deeply anesthetized with urethane (1.25 g/kg) and transcardially perfused with 0.1 M PBS followed by 4% paraformaldehyde. Brains were removed and post-fixed in the same fixative at 4°C overnight, then cryoprotected with 30% sucrose at 4°C for 1 day. Coronal brain sections (50-μm thickness) were collected with a microtome (SM2000R, Leica). The sections were permeabilized in 0.25% Triton X-100 in 0.1 M PBS (0.25% PBST) for 0.5 h, followed by blocking solution (0.1% PBST with 1% of donkey serum) for 1 h and then incubated in blocking solution with primary antibody overnight at 4°C. The primary antibodies used in the present study included: rabbit anti-Iba1 (1:1,000, 019-19741, Wako), goat anti-GFP (1:1,000, 600-101-215, Rockland). After incubation, the sections were washed 3 times in 0.1% PBST (5 min/wash) and immersed in 0.1% PBST with the corresponding secondary antibody with a dilution of 1:1000 for 3 h at room temperature. The secondary antibodies used in the present study included: donkey anti-goat 488 (A-11055, ThermoFisher), and donkey anti-rabbit 594 (711-585-152, JacksonImmunoResearch). Finally, the sections were rinsed 3 times in 0.1 M PBS (5 min/rinse) and mounted onto micro slides. Images were captured with a confocal microscope using z-stack mode with 2-μm steps under x60 magnification (FV1000, Olympus). All parameter settings for image collection such as laser power, gain, offset, zoom, scan speed, and image resolution were kept consistent between images. In general, 4–6 images were collected from each examined region for one animal, and morphological analysis was performed on 2–4 randomly selected microglia per image. For microglial density, number of microglia per area was counted for each image and compared among different conditions. For microglial morphology analyses, images containing the cell bodies of measured microglia were stacked together, and then NeuronJ (a plugin available in ImageJ, National Institutes of Health, Bethesda, MD, USA) was used to trace all the processes of each microglia. The total process length of each microglia was quantified and compared between different conditions.

### Statistics

The investigator was not blinded to group information while conducting the experiment, but was blinded during data analysis. All analysis were performed using MATLAB, results were expressed as mean ± SEM. Differences between individuals were analyzed by two-sided unpaired *t*-test, two-sided paired *t*-test, two-sided Kolmogorov–Smirnov test, or one-way ANOVA with Fisher’s *post hoc* test.

## Data availability statement

The raw data supporting the conclusions of this article will be made available by the authors, without undue reservation.

## Ethics statement

The animal study was reviewed and approved by Stony Brook Animal Care and Use Committee.

## Author contributions

HL, SG, and QX designed the experiments and wrote the manuscript. HL performed most of the experiments and data analysis. MB performed age-related morphological analysis for microglia. SS helped with data analysis. GC helped in additional analysis and proofreading. All authors contributed to the article and approved the submitted version.
