# Supplementary material for: Microglial repopulation alleviates age-related decline of stable wakefulness in mice
Source: Front Aging Neurosci. 2022 Oct 3;14:988166. doi: 10.3389/fnagi.2022.988166 (PMC9574185; doi:10.3389/fnagi.2022.988166)
Supplement: Supplementary file 1 [file Data_Sheet_1.docx]

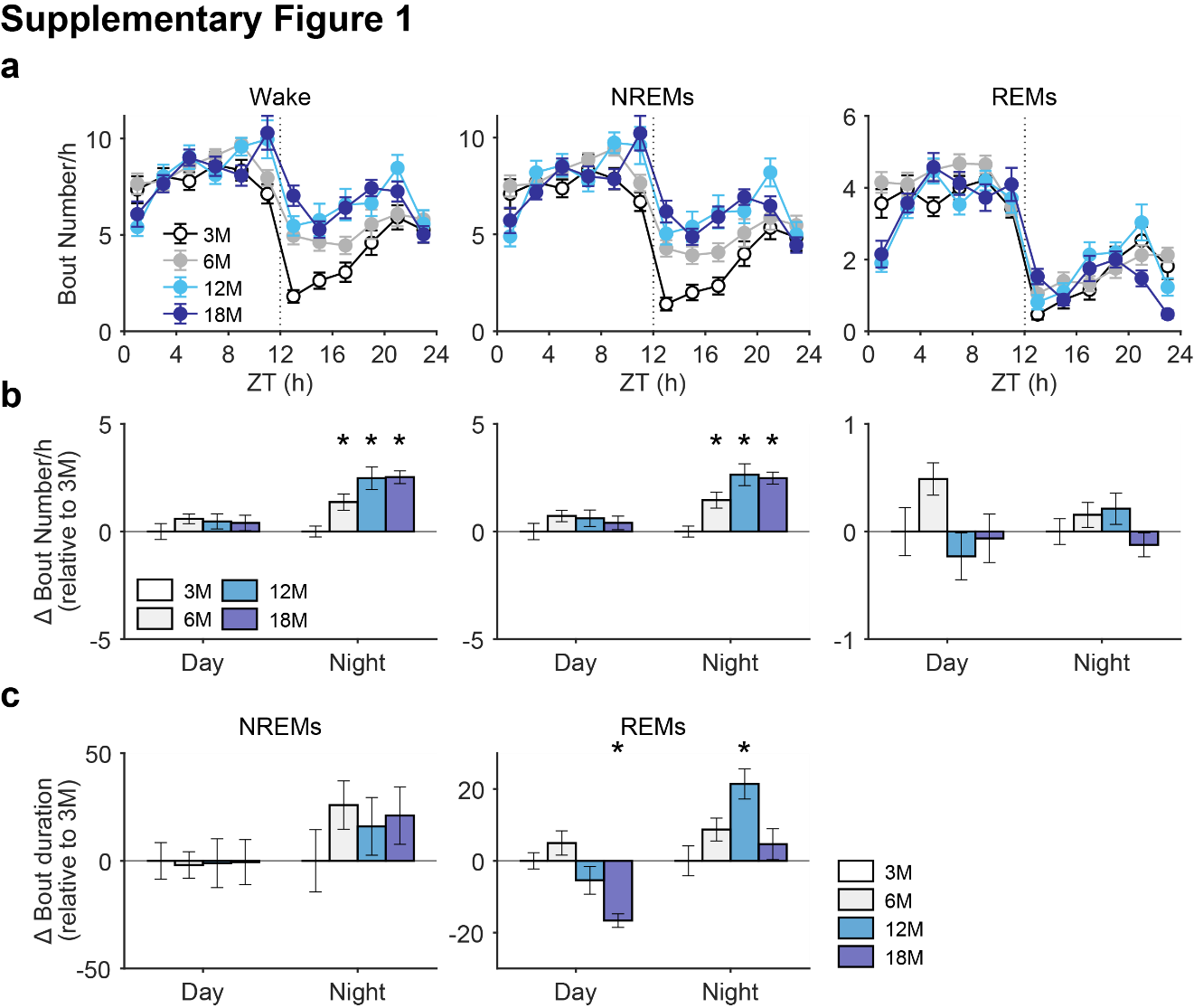


**Supplementary Figure 1. Age-related changes in bout durations and bout numbers for each brain state.**

1. Bout number of wake (left), NREMs (middle), and REMs (right) in mice with 24 hours recording at indicated ages. Data were analyzed every 2 hours. 3M, N = 16 recordings from 4 mice; 6M, N = 20 recordings from 4 mice; 12M, N = 16 recordings from 4 mice; 18M, N = 20 recordings from 4 mice.
2. Change in bout number of wake (left), NREMs (middle), and REMs (right) at day and night in mice at indicated ages. All values were normalized to data collected from 3 months old mice.
3. Change in bout duration of NREMs (left) and REMs (right) at day and night in mice at indicated ages. All values were normalized to data collected from 3 months old mice.

*, p < 0.05; two-sided unpaired t-test for a & c.


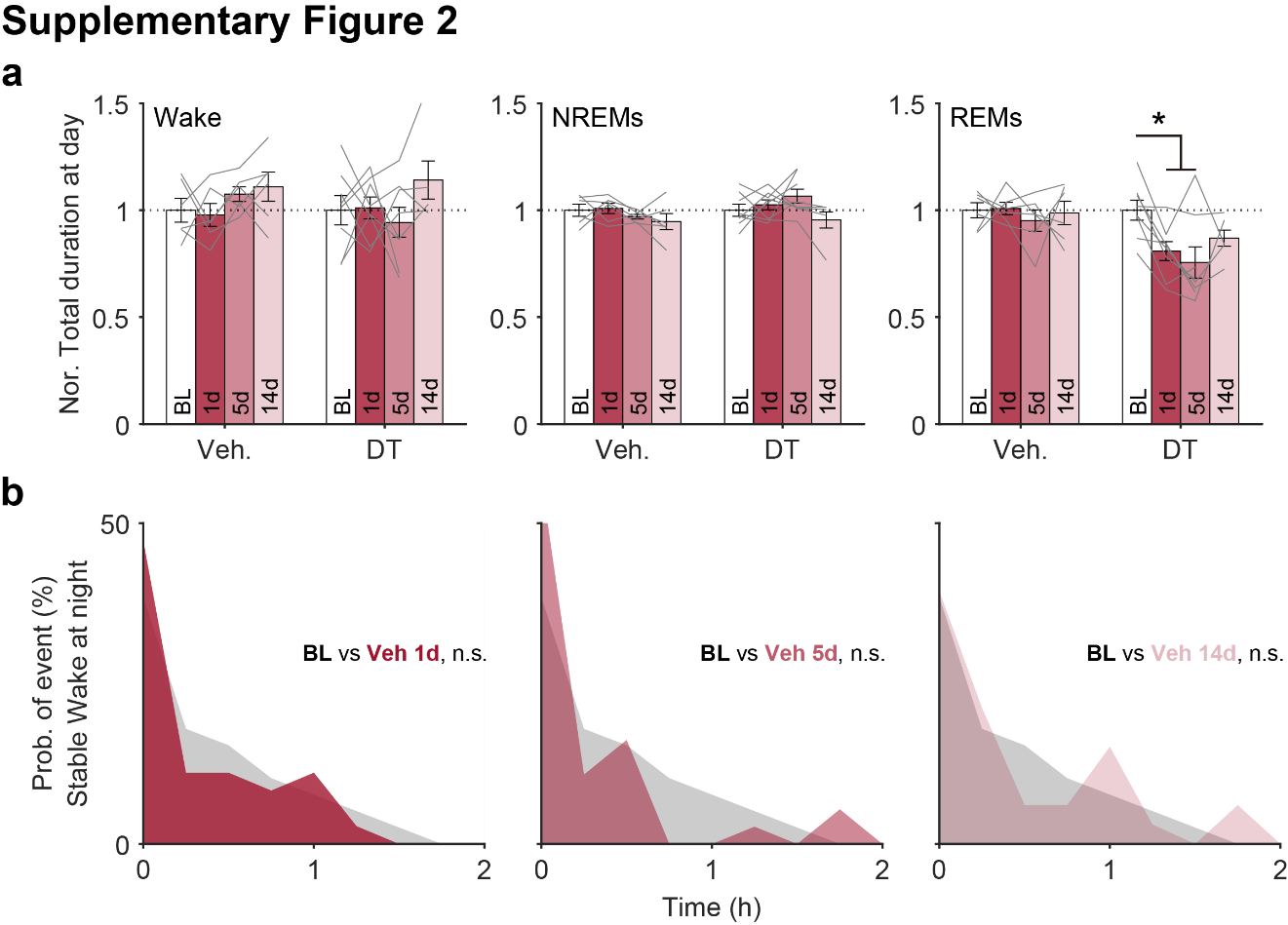


**Supplementary Figure 2. Daytime sleep architecture in mice with microglial repopulation.**

1. The normalized total duration of wake (left), NREMs (middle), and REMs (right) at daytime in mice with vehicle (Veh.) or diphtheria toxin (DT) injections. Veh., 5 mice; DT, 5 mice.
2. Distribution of duration for stable wakefulness at night between BL and day 1 (left), day 5 (middle) and day 14 (right) in control mice with vehicle injection

*, p < 0.05, two-sided paired t-test for a; two-sided Kolmogorov–Smirnov test for b.


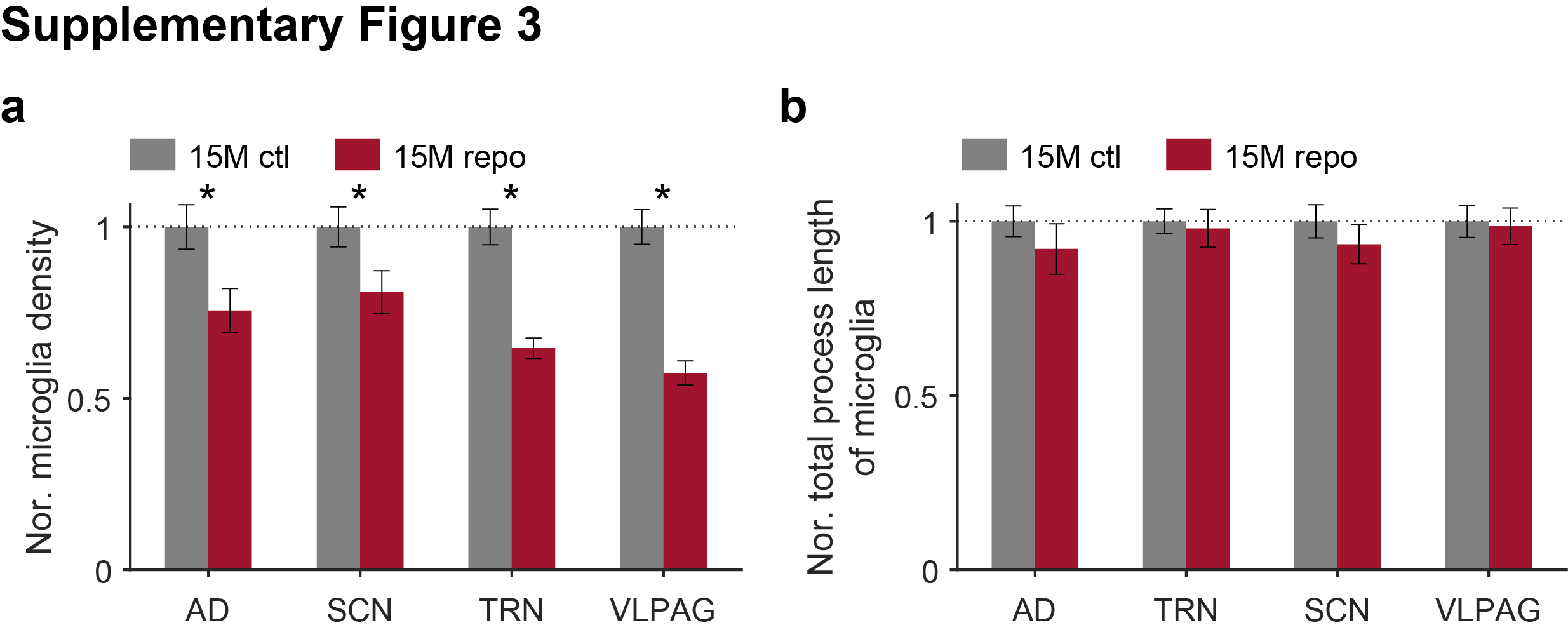


**Supplementary Figure 3. Microglia density and morphology in 15-month-old mice with microglial repopulation at adulthood.**

1. Microglia density in each brain region of 15-month-old mice with (15M repo, 5 mice) or without (15M ctl, 4 mice) microglia repopulation in adulthood. AD, anterodorsal thalamus; SCN, suprachiasmatic nucleus; TRN, thalamic reticular nucleus; VLPAG, ventrolateral periaqueductal gray.
2. Total process length of microglia in each brain region of 15-month-old mice with or without microglia repopulation in adulthood.

*, p < 0.05, two-sided paired t-test for a& b
